# Giant cell tumor of the pubic bone with concomitant enchondroma: a case report

**DOI:** 10.3389/fmed.2026.1843522

**Published:** 2026-05-12

**Authors:** Hongjing Sun, Minghao Gao, Hong Yu, Xiaohui Feng

**Affiliations:** 1 Department of Pathology, The Third Hospital of Hebei Medical University, Shijiazhuang, China; 2 School of Basic Medicine, Hebei Medical University, Shijiazhuang, China; 3 Department of Radiology, The Third Hospital of Hebei Medical University, Shijiazhuang, China

**Keywords:** collision tumor, enchondroma, giant cell tumor of bone, immunohistochemistry, pubic bone

## Abstract

Giant cell tumor of bone (GCTB) is a locally aggressive, intermediate-type neoplasm, accounting for approximately 4%−5% of primary bone tumors, typically arising in the metaphysis of long bones. Enchondroma is a common benign cartilaginous tumor, frequently localized in the short tubular bones of the hands and feet. The coexistence of these two distinct histopathological entities at the same anatomical site is exceedingly rare. We report a case of a 36-year-old female who presented with left hip pain after exertion for a duration of 2 weeks. Imaging studies revealed an osteolytic, expansile lesion in the left pubic bone, suggestive of GCTB. Histopathological examination of the resected specimen demonstrated two distinct morphological patterns: areas characteristic of GCTB (immunopositive for H3.3 G34W) coexisting with areas of enchondroma. This coexistence is exceptionally uncommon in bone tumors, particularly at the pubic location. Notably, the GCTB component exhibited extraosseous soft tissue extension and identifiable intravascular tumor thrombi, with a Ki-67 proliferation index reaching 40%, indicating a high aggressive potential. Corroborated by a review of the literature, this case emphasizes the critical role of histopathological evaluation in diagnosing bone tumors and discusses the diagnostic approach and clinical implications of such a rare tumor combination.

## 1 Introduction

Giant cell tumor of bone (GCTB) is a relatively common primary bone neoplasm, representing 4%−5% of all primary bone tumors. It predominantly affects individuals in the third and fourth decades of life, with a slight female predilection. The typical localization is the metaphysis of long bones, most commonly the distal femur, proximal tibia, and distal radius. Pelvic involvement is infrequent, with an incidence ranging from approximately 1.5%−6.1% (1, 2). GCTB is classified as an intermediate-type tumor with local aggressiveness. Although GCTB lacks a high intrinsic metastatic potential, a small subset of cases can develop pulmonary metastases (3, 4). Recent molecular studies have identified driver mutations in the H3F3A gene in over 90% of GCTB cases, resulting in the substitution of glycine with tryptophan at position 34 of histone H3.3 (H3.3 G34W). This discovery has provided a highly specific immunohistochemical marker for the diagnosis of GCTB (5–7).

Enchondroma is the second most common benign cartilaginous tumor (following osteochondroma), accounting for approximately 3% of all bone tumors and 13% of benign bone neoplasms. It is most frequently observed in individuals aged 10–30 years, typically involving the short tubular bones of the hands and feet, followed by the femur and humerus; pelvic localization is uncommon (8–10). Histologically, enchondroma is composed of lobules of mature hyaline cartilage. The chondrocytes are sparse, with small, hyperchromatic nuclei; binucleated cells are rare, and mitotic figures are absent (11, 12).

The simultaneous occurrence of two different histological types of tumors at the same anatomical site is exceptionally rare in bone oncology. A literature search reveals only two reported cases of GCTB coexisting with enchondroma (13). This article reports a case of GCTB of the pubic bone associated with an enchondroma, detailing its histomorphological features, immunophenotype, and key differential diagnoses, aiming to enhance the understanding of this rare tumor combination.

## 2 Case presentation

### 2.1 Clinical data

A 36-year-old female presented with left hip pain following exertion for 2 weeks. The pain was exacerbated by activity and relieved by rest, did not disturb nocturnal sleep, and was not associated with systemic symptoms such as fever or night sweats. Her past medical history was unremarkable, with no history of trauma or family history of neoplasms. Physical examination revealed no discernible mass in the pubic symphysis region, no local tenderness, no palpable mass, and normal local skin temperature. The range of motion in both hip, knee, and ankle joints was unrestricted.

### 2.2 Auxiliary examinations

#### 2.2.1 Imaging findings

**Figure 1:**
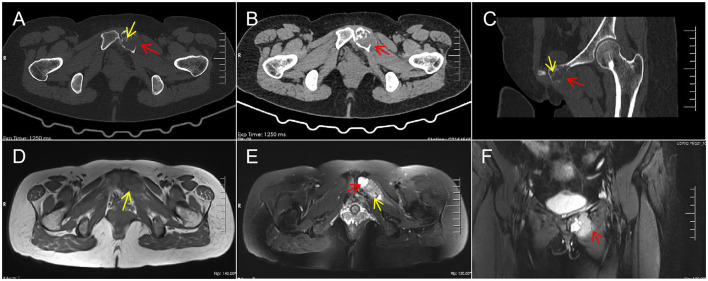
CT revealed: **(A, C)** Osteolytic bone destruction of the left pubic bone with expansile remodeling, cortical thinning and disruption (red arrows), and intralesional bony septa (yellow arrows) (bone window); **(B)** Surrounding soft tissue mass (red arrow) (soft tissue window). MRI revealed: **(D)** T1WI demonstrated hypointense signal (yellow arrow). **(E)** T2WI revealed heterogeneous signal intensity, with the main portion of the lesion demonstrating iso- to hyperintense signal (yellow arrow), and a nodular area of more pronounced hyperintensity anteriorly (red arrow). **(F)** T2WI demonstrated involvement of the surrounding soft tissue (red arrow).

Computed Tomography (CT) revealed (Figure 1): osteolytic bone destruction of the left pubic bone with expansile remodeling, cortical thinning and disruption, heterogeneous attenuation, intralesional bony septa, surrounding soft tissue swelling, and no evidence of periosteal reaction.

Magnetic Resonance Imaging (MRI) revealed (Figure 1): expansile bone destruction in the left pubic bone, demonstrating hypointense signal on T1-weighted imaging (T1WI) and heterogeneous signal on T2-weighted imaging (T2WI). The main portion of the lesion predominantly exhibited iso- to hyperintense signal, with a nodular area of more pronounced hyperintensity anteriorly on T2WI. Mild edema was noted in the adjacent bone marrow, with involvement of the surrounding soft tissue. The imaging findings were highly suggestive of giant cell tumor of bone.

Chest CT and whole-body bone scintigraphy showed no evidence of distant metastasis or multifocal disease.

#### 2.2.2 Laboratory findings

Serum calcium level was 2.46 mmol/L (reference range: 2.22–2.52 mmol/L). Serum alkaline phosphatase and serum phosphorus levels were within normal limits.

### 2.3 Surgical treatment

Surgical treatment After completing preoperative preparations and ruling out contraindications, the patient underwent resection of the left pubic bone tumor under general anesthesia (laryngeal mask airway) combined with regional nerve block. Intraoperatively, bone destruction of the left pubic symphysis and superior pubic ramus was observed, forming a soft tissue mass. The superior and inferior pubic rami were exposed through normal tissue planes outside the mass. Osteotomies of the superior and inferior pubic rami and the pubic symphysis were performed using an ultrasonic bone scalpel. The tumor was carefully retracted and completely resected along with a cuff of surrounding normal tissue from its deep aspect.

### 2.4 Pathological examination

**Figure 2:**
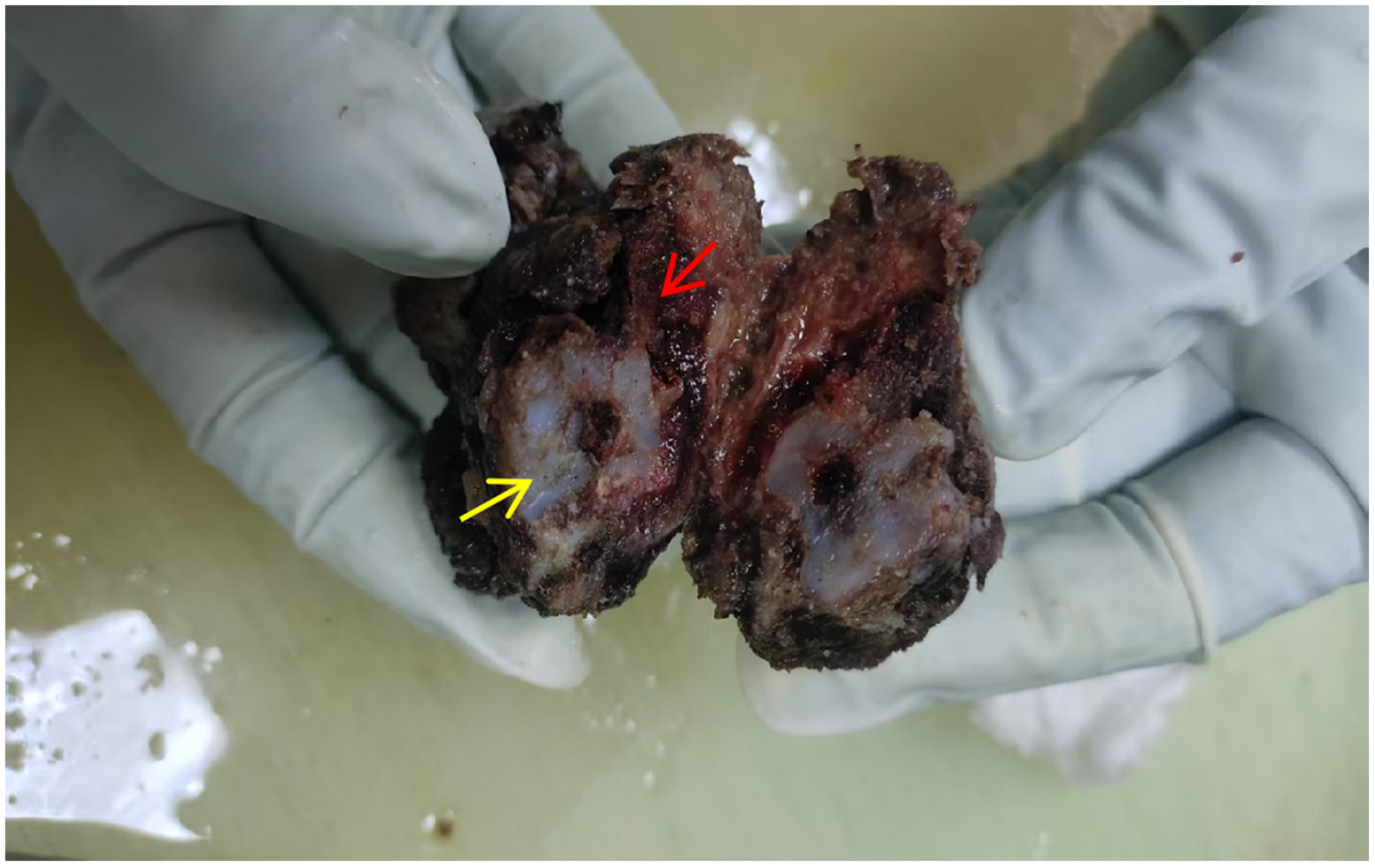
The cut surface exhibits a biphasic appearance (red arrow: GCTB region; yellow arrow: enchondroma region).

Gross findings: The submitted tumor specimen measured approximately 4 **×** 3.5 **×** 3 cm. The cut surface showed a biphasic appearance: some areas were gray-white, firm, translucent, and lobulated with a cartilaginous appearance; other areas were gray-white to gray-red and firm in consistency (Figure 2).

**Figure 3:**
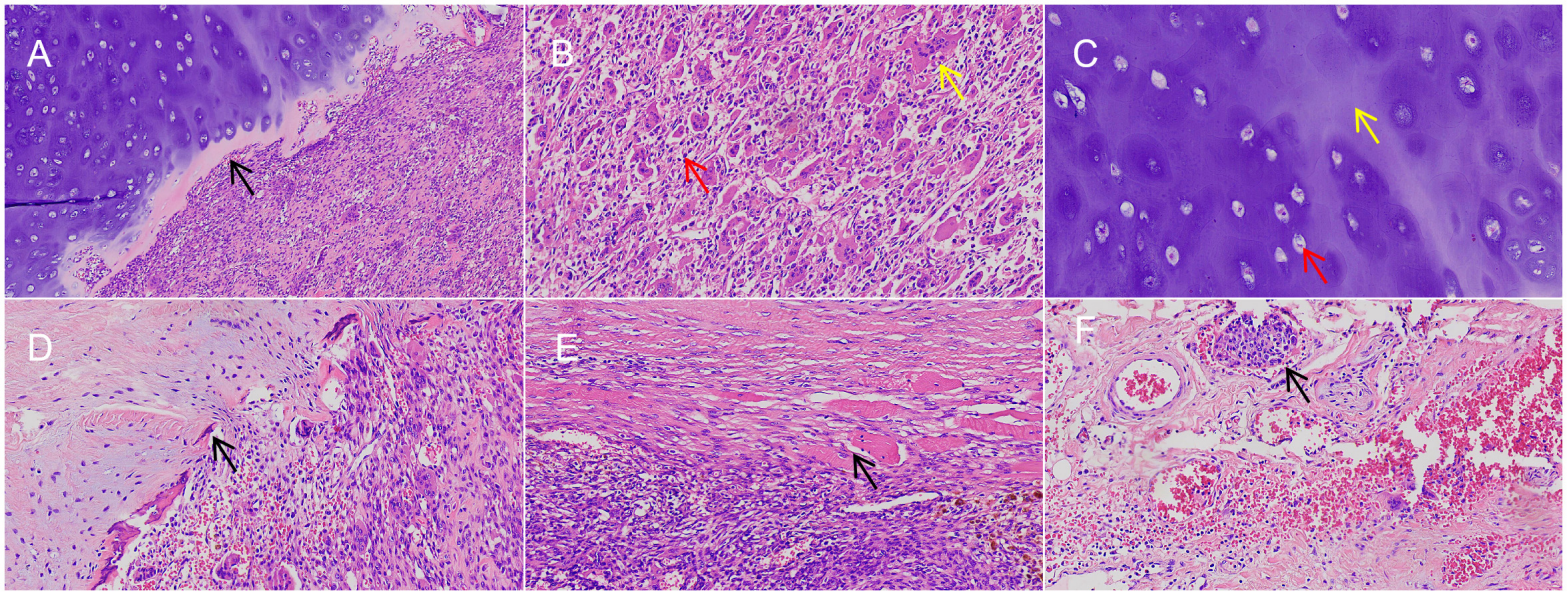
**(A)** Two distinct morphological areas with a sharp border and no transitional zone (black arrow; HE, × 100). **(B)** GCTB region (red arrow: mononuclear stromal cells; yellow arrow: multinucleated osteoclast-like giant cells; HE, × 200). **(C)** Enchondroma region (red arrow: chondrocytes; yellow arrow: cartilaginous matrix; HE, × 200). **(D)** Cortical bone breakthrough (black arrow; HE, × 200). **(E)** Extraosseous soft tissue extension (black arrow; HE, × 200). **(F)** Intravascular tumor thrombus (black arrow; HE, × 200).

Microscopic findings: Microscopically, the tumor consisted of two distinct morphological areas with a sharp border and no transitional zone between them (Figure 3). Area 1 (GCTB region): composed of diffusely distributed mononuclear stromal cells and uniformly scattered multinucleated osteoclast-like giant cells. The mononuclear cells were ovoid or short spindle-shaped with eosinophilic cytoplasm and ovoid, vesicular nuclei containing small nucleoli. The multinucleated giant cells were large with abundant cytoplasm and contained dozens of nuclei resembling those of the mononuclear cells (Figure 3). No significant nuclear atypia or atypical mitotic figures were observed. This component invaded the extraosseous soft tissue, and tumor cell nests were identified within small blood vessels at the tumor periphery, confirming intravascular tumor thrombi (Figure 3). Area 2 (Enchondroma region): composed of lobulated growth of hyaline cartilage, with thin rims of reactive bone surrounding the lobules. The chondrocytes were sparse, located within lacunae, and had small, hyperchromatic, pyknotic nuclei; occasional binucleated cells were seen, but no mitotic figures were identified. The cartilage matrix was uniformly basophilic (Figure 3). This region showed no cellular atypia, myxoid change, or entrapment of host bone, consistent with a diagnosis of enchondroma.

**Figure 4:**
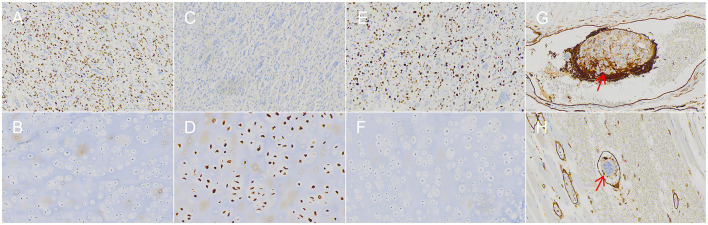
**(A, B)** The GCTB region is positive for H3.3 G34W, while the enchondroma region is negative for H3.3 G34W (IHC, × 200). **(C, D)** The GCTB region is negative for S-100, while the enchondroma region shows positive S-100 immunostaining (IHC, × 200). **(E, F)** The GCTB region demonstrates a Ki-67 proliferation index of 40% in hot spots, while the enchondroma region shows a Ki-67 index of < 1% (IHC, × 200). **(G, H)** Intravascular tumor thrombus: CD31 immunohistochemical staining highlights the vascular endothelium, clearly demonstrating the tumor thrombus within the vessel lumen (red arrow indicates the tumor thrombus, IHC, × 200).

Immunohistochemical profile: Immunohistochemical staining was performed separately for the two regions, yielding the following results (Figure 4): GCTB region: H3.3 G34W (+) (strong nuclear positivity in mononuclear stromal cells, negative in multinucleated giant cells), H3K36M (-), CD56 (+), P63 (+), CD163 (+), SATB2 (+), Ki-67 (40%+, in hot spots), S-100 (-), SMA (-). Enchondroma region: S-100 (+) (nuclear and cytoplasmic positivity in chondrocytes), H3.3 G34W (-), P63 (-), Ki-67 (< 1%+). Intravascular tumor thrombus (Figure 4): CD31 immunostaining highlighted the vascular endothelium, clearly demonstrating the tumor thrombus within the vessel lumen.

Pathological diagnosis: Based on the comprehensive pathological features, the final diagnosis was: giant cell tumor of the left pubic bone, with extension into extraosseous soft tissue and presence of intravascular tumor thrombi; associated with a peripheral enchondroma. Both the soft tissue margins and the bone resection margins are free of tumor.

### 2.5 Follow-up

The patient recovered well postoperatively with satisfactory wound healing. Regular follow-up was conducted every 3 months, including local CT and MRI. As of 1 year post-surgery, there is no evidence of disease recurrence or pulmonary metastasis.

## 3 Discussion

Pelvic bone tumors account for only 3%−4% of primary bone neoplasms, and both GCTB and enchondroma occurring in the pelvis are uncommon (2, 8, 9, 14, 15). Imaging is a crucial auxiliary tool for diagnosing GCTB. Typical CT findings for GCTB include an osteolytic lesion with bone remodeling, cortical thinning, and endosteal scalloping; periosteal reaction is relatively uncommon. On MRI, lesions typically show low/intermediate signal intensity on T1WI and heterogeneous high signal intensity on T2WI due to the presence of fibrous components, cystic areas, and hemosiderin deposition (1). The imaging features of the present case were consistent with GCTB. However, a definitive diagnosis of GCTB still relies on histopathological examination. Histologically, GCTB is characterized by diffuse proliferation of mononuclear stromal cells uniformly admixed with numerous osteoclast-like multinucleated giant cells, often accompanied by hemorrhage and hemosiderin deposition. The present case met the pathological diagnostic criteria for GCTB. In recent years, the application of H3.3 G34W immunohistochemistry has greatly improved the diagnostic accuracy and specificity for GCTB. Multiple studies have confirmed that H3.3 G34W IHC has a sensitivity of approximately 88%−96% and specificity nearing 100% for diagnosing GCTB (4–7). The strong positivity for H3.3 G34W in the GCTB region of this case further supports the diagnosis. The diagnosis of enchondroma requires differentiation from low-grade chondrosarcoma. Imaging can assist in differentiating cartilaginous tumors, but differentiating enchondroma from low-grade chondrosarcoma can be challenging even with CT and MRI (16, 17). Histopathological diagnosis is key to distinguishing enchondroma from low-grade chondrosarcoma. Histologically, enchondroma consists of lobules of mature hyaline cartilage with sparse chondrocytes showing small, hyperchromatic nuclei; binucleated cells are rare, and mitotic figures are absent. Low-grade chondrosarcoma, in contrast, exhibits infiltrative growth, entrapment of host lamellar bone, increased cellularity, nuclear atypia, and may show myxoid change (11, 12). The enchondroma region in this case showed no infiltrative growth, myxoid change, increased cellularity, or nuclear atypia, and the immunophenotype was consistent with a benign lesion.

A collision tumor refers to the coexistence of two distinct histological types of tumors at the same anatomical site, with a sharp border and no transitional zone between them. Collision tumors are extremely rare in bone. Ruckstuhl et al. (13) first systematically reported a case series of GCTB coexisting with other primary bone tumors in 1980. Among the three cases, two were GCTB associated with enchondroma (one located at the distal femur plus femoral shaft, and the other at the proximal tibia plus proximal fibula), and the third was GCTB associated with non-ossifying fibroma (NOF). The authors proposed three possible mechanisms: chance occurrence, induction of one tumor by the other, or shared local factors (e.g., local growth stimulation) promoting the development of both tumors. However, all cases in this series occurred around the knee joint (long bones of the extremities), with the two tumor types located in different bones or different regions of the same bone, rather than being adjacent within the same bone (13). Subsequently, no reports of GCTB and enchondroma occurring within the same bone as a collision tumor have been documented in the domestic or international literature. This article reports the first case of GCTB combined with enchondroma in the left pubic bone, with both tumor components coexisting within the same bone but lacking a transitional zone. In the present case, two completely different histological types, GCTB and enchondroma, appeared within the same bone lesion, with clear demarcation and no transition. The immunophenotypes were distinct: the GCTB area was H3.3 G34W (+) and S-100 (-); the cartilaginous area was H3.3 G34W (-) and S-100 (+). This meets the diagnostic criteria for a collision tumor and enhances the understanding of this rare combination.

GCTB is locally aggressive and classified as an intermediate-type tumor. Although it lacks intrinsic metastatic potential, pulmonary metastasis occurs in 1%−9% of cases (3, 4, 18, 19). The presence of vascular invasion suggests a significantly increased risk of distant metastasis. Literature reports indicate that pathologically confirmed vascular invasion in primary GCTB can be associated with progressive pulmonary metastasis (20). Ruckstuhl et al. (13) emphasized that en bloc resection is mandatory for GCTB. Among the three cases they reported, the first two underwent primary en bloc resection and remained free of recurrence (with a maximum follow-up of 4 years). The third case initially underwent curettage alone, which led to local recurrence, ultimately necessitating arthrodesis and even amputation. This experience underscores the importance of achieving R0 resection, especially for GCTB occurring in weight-bearing bones or anatomically complex sites. In the present case, en bloc tumor resection was performed using an ultrasonic bone scalpel. At one-year follow-up, no recurrence or pulmonary metastasis was observed, confirming the effectiveness of the en bloc resection strategy. However, pulmonary metastasis of GCTB can occur several years or even more than a decade after surgery, with isolated cases of longer intervals of as much as 24 years having been reported (18, 19). In this case, the pathology report clearly stated “intravascular tumor thrombi,” and the Ki-67 proliferation index was as high as 40%. Although the tumor histologically appears as typical GCTB, these findings suggest a biological behavior with high invasive potential. Therefore, the absence of metastasis at 1 year does not rule out long-term risk. Long-term (at least 5–10 years), regular imaging follow-up—including local CT, MRI, and whole-body bone scintigraphy—should be maintained. The current disease-free survival status of this patient not only validates the efficacy of the surgical strategy but also highlights the critical importance of adhering to long-term, regular imaging follow-up for GCTB patients with vascular invasion.

H3.3 G34W IHC has become an essential tool for the diagnosis and differential diagnosis of GCTB. Studies have shown that this antibody shows nuclear positivity in the mononuclear stromal cells of GCTB, while the osteoclast-like multinucleated giant cells are negative, a characteristic staining pattern. In terms of differential diagnosis, H3.3 G34W can effectively distinguish GCTB from other giant cell-rich bone lesions, such as chondroblastoma (H3K36M+), non-ossifying fibroma, aneurysmal bone cyst, and giant cell reparative granuloma, all of which are negative for H3.3 G34W (4, 6). The H3.3 G34W positivity in this case provides molecular-level evidence supporting the GCTB diagnosis. It is noteworthy that approximately 5%−10% of GCTBs may harbor variant mutations other than H3.3 G34W, such as G34V, G34R, or G34L. These cases would show negative staining with routine H3.3 G34W IHC. For cases with high clinical suspicion of GCTB but negative H3.3 G34W staining, additional IHC for H3.3 G34V or G34R, or H3F3A gene sequencing, should be considered (7).

The biphasic histology of this case requires differentiation from the following entities:

1. GCTB with secondary cartilage differentiation: Very rare GCTBs may exhibit focal cartilaginous matrix formation. However, such cartilaginous areas typically show transition zones with the typical GCTB areas, and the mononuclear cells within the cartilaginous areas may still express H3.3 G34W. In the present case, the two areas were sharply demarcated without a transition zone, which does not support a secondary change (6).

2. Chondroblastoma: Typically located in the epiphysis, composed of chondroblasts and multinucleated giant cells. However, chondroblasts are polygonal with nuclear grooves and express S-100 and H3K36M immunohistochemically, but not H3.3 G34W, distinguishing it from GCTB (5, 6).

3. Chondrosarcoma with osteoclast-like giant cell reaction: Low-grade chondrosarcomas may be accompanied by reactive osteoclast-like giant cells at the periphery. However, the giant cells are unevenly distributed, and the chondrosarcomatous areas show infiltrative growth, entrapment of host bone, increased cellularity, and possible myxoid change, which are inconsistent with the findings in this case (12).

4. Brown tumor of hyperparathyroidism: Brown tumors are typically associated with hypercalcemia, hypophosphatemia, and elevated parathyroid hormone (PTH) levels. In the present case, the serum calcium level was 2.46 mmol/L (within the normal range), with no evidence of hyperparathyroidism. Histopathologically, brown tumors are characterized by reactive aggregation of giant cells and lack H3.3 G34W expression (4).

5. NOF: In NOF, giant cells are predominantly aggregated in areas of hemorrhage or hemosiderin deposition rather than being uniformly distributed. In the present case, positivity for H3.3 G34W and SATB2 effectively excludes the diagnosis of NOF (5, 6).

## 4 Conclusion

This article reports a rare case of giant cell tumor of the pubic bone associated with an enchondroma. Pathological examination not only confirmed the coexistence of the two tumor components but also revealed the presence of intravascular tumor thrombi and high proliferative activity within the GCTB component. These findings indicate a high invasive potential for this tumor, necessitating the establishment of a long-term, strict follow-up plan focused on monitoring for pulmonary metastasis. This case underscores the critical importance of pathological examination in the diagnosis of bone tumors, particularly the assessment of vascular invasion and the identification of rare histological combinations, which are invaluable for patient prognosis and treatment strategy determination.

## Data Availability

The original contributions presented in the study are included in the article/supplementary material, further inquiries can be directed to the corresponding author.
